# Cuticular Pegs near Wing Bases in Aphids of the Subfamily Eriosomatinae Kirkaldy, 1905 s. str. (Insecta, Aphididae)

**DOI:** 10.3390/insects16121200

**Published:** 2025-11-26

**Authors:** Agnieszka Nowińska, Łukasz Depa

**Affiliations:** Institute of Biology, Biotechnology and Environmental Protection, Faculty of Natural Sciences, University of Silesia in Katowice, Bankowa 9, 40-007 Katowice, Poland; agnieszka.nowinska@us.edu.pl

**Keywords:** proprioception, chordotonal organ, sensilla, locomotion, flight

## Abstract

**Simple Summary:**

Effective flight plays a crucial role for aphids. Their alate morphs need to search and find appropriate host plant species, as many aphid species are specialized to feed on particular plant species and genera. Flying needs to be effective and requires many specialized structures and sensory organs to ensure their proper function. Here, we present the first data on the cuticular pegs located at the base of the aphid forewings. Similar structures have been studied in some other groups of insects, but not in aphids. These pegs, or rods, are located on the special exoskeletal plate, tegula, and seem to play the role of sensory organs which regulate the bending of the wings during flight.

**Abstract:**

Aphids have two general types of morphs: alate and apterous. While apterous morphs exploit existing local resources, the alate morphs disperse in search of proper host plant species for settlement and reproduction. Acquiring information on the position of the body and wings requires a set of various sensory organs, which provide visual, olfactory, and proprioceptive information. The latter ones are provided through various sensilla, also located on the wings. Here, we present data on the cuticular pegs located on the membranous part of the tegula in alate aphids of the subfamily Eriosomatinae. These cuticular pegs, located on the distal part of the tegula, seem to have a sensory function, but their functional mode is unknown. It is hypothesized that they play either a mechanical function during movement of the wing, or are a part of the chordotonal organ, often located near the base of the wing in insects.

## 1. Introduction

Insect wings possess a sensory apparatus that, among other functions, helps with the movement of the wings and body stabilization during flight [1]. An array of different sensory structures (sensilla) are found on the surface of the wings [2].

Mechanoreceptors (which are responsible for detecting touch, pressure, stretching, vibration or other movements that alter their position) are mostly present in the form of hairs or discs on an insect’s body. The hairs are usually tapered, with a ribbed surface. The discs are round or oval, with a singular molting pore in the middle. Those mechanoreceptors can be distinguished due to possessing a well pronounced socket, with a thin membrane connecting the cuticle of the body with the cuticle of the sensillum. This gives the sensillum a greater movement range [3,4,5,6]. On insect wings, the usually described mechanoreceptors are the discs called campaniform sensilla. They react to pressure so they can measure positional changes at wing joints and deformations of the wing blade [7]. Hair sensilla also appear on the wings. They react to external forces like touch or air current. However, only some of the hairs are sensory structures. Many are not innervated or are just microtrichia. The true mechanoreceptors are positioned at the margins of the wings [8,9]. They are believed to play a role in detecting airborne vibrations and controlling wingbeats [10]. Other types of mechanoreceptors that are found on wings include stretch and chordotonal proprioceptors. They are structures located exclusively on the hinge between the wing and the thorax [11]. Stretch receptors are responsible for regulating wing beat rhythmicity, by detecting stretching, compression, and muscle tension, and chordotonal receptors, usually clustered into so-called scoloparia, encode inputs during flight [2,3]. In some insects, these chordotonal organs on the wing’s hinge have been transformed into exteroceptors sensitive to sound pressure [12].

Apart from mechanoreceptive sensilla, possible chemo- and thermoreceptive sensilla are found on insects’ wings.

Chemoreceptors appear as gustatory tip–pore hair sensilla and are present along the wing margin [13]. Their biological role is still poorly understood. However, their function has been experimentally proven, and it has been hypothesized that pollinators might use them in order to probe nectar sources without having to land on the flower [14].

Thermoreceptors may detect temperature changes in the environment and allow for thermoregulation, host and mate detection, or other specialized functions [3]. The existence of thermoreceptors on the wing of heliotherm butterflies was argued by Schmitz and Wasserthal [15], by scanning the wing veins with a calibrated light beam. A slow wing-closing reaction is hypothesized to be a protective mechanism against overheating, which would confirm the presence of thermoreceptors.

The base of the wing, together with the membrane, forms a complex structure responsible for the ability to fly [16,17,18]. It comprises several elements that cooperate, like axillary sclerites and muscles [19]. The structure of interest for this study—the tegula—is a scale-like lobe that overlaps the base of the forewing in some insects. It is usually represented by a small pad or lobe at the anterior root of the wing base [20]. Different functions have been suggested for the tegula and the receptors found on it. In locusts, where the structure has been extensively studied, the tegula is believed to directly control flight muscles by initiating an upstroke when electrically stimulated [21]. In some Lepidoptera, the tegula takes part in stridulation [22]. The presence of sensilla on the tegula has been studied mostly in Acrididae and Calliphoridae [18,23,24]. Those studies showed the presence of mechanoreceptive sensilla, mostly campaniform sensilla, hair-like sensilla, and chordotonal organs. Knyazeva [25] also documented a multiterminal stretch receptor complex.

For trophically specialized herbivorous aphids (Insecta, Hemiptera, Aphidomorpha), flight ability is crucial in finding proper host plant species. In many species’ life cycle, this process occurs twice: in spring, when alate females search for secondary hosts where they reproduce parthenogenetically, and in autumn, when alate sexuparous morphs search for primary hosts where they give birth to oviparous females and males, e.g., in the aphid subfamily Eriosomatinae Kirkaldy, 1905 s. str. (excluding Pemphiginae) [26,27]. Due to the variable abundance and distribution of host plant species, as well as variable weather conditions during flight, finding a proper host plant seems to be a demanding task [28].

Presently, no studies exist on the tegula of aphids. Most existing studies on aphid wing sensilla comprise data on morphology and distribution of the trichoid and campaniform sensilla placed on the forewings [29,30,31]. Here, we present the first data on the presence of structures on the base of the aphid’s forewing, recognized as a potential chordotonal organ.

## 2. Materials and Methods

Three species from the subfamily Eriosomatinae s. str. (excluding Pemphiginae Herrich-Schaeffer, 1854), [*Favret, C.* *[2025]. Aphid Species File. [12.11.2025]. http://Aphid.SpeciesFile.org*] were used for this study. For scanning electron microscopy and light microscopy, newly collected specimens were used. For their determination, three specimens from each collection were mounted, and deposited in the entomological collection of the Faculty of Natural Sciences of the University of Silesia in Katowice (DZUS) under the following labels and accession numbers:

 

*Eriosoma ulmi* (Linnaeus, 1758) from *Ulmus laevis*, Katowice, leg. & det. Ł. Depa, 19.05.2025, alate fundatrigenia, 3 specimens, DZUS 19-5-25-2-3—from this collection additional 8 specimens for SEM study.

 

*Tetraneura ulmi* (Linnaeus, 1758) from *Ulmus laevis*, Katowice, leg. & det. Ł. Depa, 15.05.2025, alate fundatrigenia, 3 specimens, DZUS 15-5-25—from this collection additional 5 specimens for SEM study.

 

*Tetraneura nigriabdominalis* (Sasaki, 1899) from *Digitaria* sp., Katowice, leg. & det. Ł. Depa, 04.09.2024; sexupara, 3 specimens, DZUS 9-24-B9—from this collection additional 2 specimens for SEM study

 

Additionally, only for light microscopy studies, the following material, already deposited in the entomological collection as mounted specimens on microscopic slides, was examined;

 

*Eriosoma ulmi* (Linnaeus, 1758) from *Ulmus laevis*, Toszek, leg. & det. Ł. Depa, 17.10.2022; sexupara, 4 specimens, DZUS 17.10.22.1.3.

 

*Eriosoma ulmi* (Linnaeus, 1758) from *Ulmus laevis*, Toszek, leg. & det. Ł. Depa, 17.10.2022; sexupara, 3 specimens, DZUS 17.10.22.2.3.

 

*Eriosoma ulmi* (Linnaeus, 1758) from *Ulmus laevis*, Dobieszowice, leg. & det. Ł. Depa, 07.10.2023; sexupara, 4 specimens, DZUS 7.10.23.

 

*Tetraneura ulmi* (Linnaeus, 1758) from *Lolium perenne*, Rogoźnik, leg. & det. Ł. Depa, 11.09.2024; sexupara, 3 specimens, DZUS 29.24.1.2.

 

*Tetraneura ulmi* (Linnaeus, 1758) from *Ulmus laevis*, Toszek, leg. & det. Ł. Depa, 17.10.2022; sexupara, 2 specimens, DZUS 17.10.22.3.3.

 

*Tetraneura ulmi* (Linnaeus, 1758) from *Ulmus glabra,* Skała, Ojców National Park, quarry, leg. & det. B. Osiadacz, 04.07.2005; alate fundatrigenia, 2 specimens, DZUS SA02-414-03-031

 

*Tetraneura ulmi* (Linnaeus, 1758) from *Poa annua*, Piekary Śląskie, leg. & det. Ł. Depa, 09.09.2003. Sexupara, 1 specimen, DZUS SA02-414-03-022

 

*Tetraneura ulmi* (Linnaeus, 1758) from *Ulmus campestris*, Slovakia, Šur, district Bratislava, Panonski Haj, leg. & det. A. Czylok, 26.06.1987, sexupara, 1 specimen, DZUSSA02-414-03-011

 

*Tetraneura ulmi* (Linnaeus, 1758) from *Ulmus campestris*, Slovakia, Šur, district Bratislava, Panonski Haj, leg. & det. A. Czylok, 26.06.1987, sexupara, 3 specimens, DZUSSA02-414-03-012

### 2.1. Light Microscopy

For light microscopy, newly collected specimens of each species were mounted on microscopic slides in a Berlese medium, following traditional aphidological procedures [26,32]. The studied structures were analyzed using a Nikon Eclipse E600 light microscope and photographed with a Nikon DS-Fi2 camera (Nikon Corporation, Tokyo, Japan) in the Laboratory of Insect Morphology and Anatomy, Faculty of Natural Sciences, University of Silesia in Katowice. Software used includes the Nikon NIS-Elements version 4.20 (Nikon Instruments Inc., Tokyo, Japan). The number of visible pegs on the forewing tegulas was counted, when the tegula was visible. During the mounting process, the tegula as well as the wing base are often hidden under the thorax, and these structures were not visible in all specimens. The number of specimens available for studies and the number of available (clearly visible) tegulas are presented in Table 1.

### 2.2. Scanning Electron Microscopy

For scanning electron microscopy, 8 specimens of *Eriosoma ulmi*, 5 specimens of *Tetraneura ulmi*, and 2 specimens of *Tetraneura nigriabdominalis* were studied. The prepared samples were analyzed at the scanning microscopy laboratory of the Faculty of Natural Science, Institute of Biology, Biotechnology and Environmental Protection of the Silesian University in Katowice (Katowice, Poland). For SEM imaging, the material stored in 70% ethanol was treated with chloroform for 48 h. Samples were then dehydrated in a series of graded ethanol solutions (80%, 90%, 96%, 99.8%) and dried in a Leica EM CPD300 critical point dryer (Leica Microsystems, Vienna, Austria). Dried samples were mounted on aluminum stubs using double-sided adhesive carbon discs and sputter-coated with a 30 nm gold layer in a Quorum 150 T ES Plus sputter coater (Quorum Technologies Ltd., Laughton, East Sussex, UK). Prepared samples were analyzed with the use of a Phenom XL (Phenom-World BV, Netherlands)—general imaging—and Hitachi UHR FE-SEM SU8010 (High Technologies, Tokyo, Japan)—high magnification imaging—scanning electron microscopes. Figures were made and edited with the use of Paint.net 5.1.2 (dotPDN, LLC, Kirkland, WA, USA).

**Figure 1:**
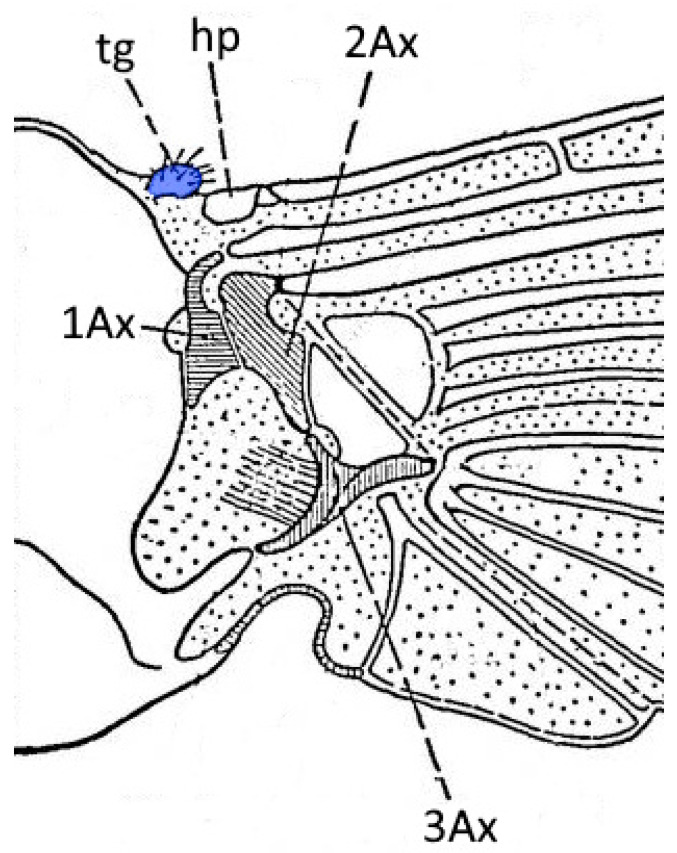
Model of insects wing base (after Snodgrass 1935 [20], modified). Abbreviations: hp—humeral plate (basicosta), tg—tegula, Ax—axillary sclerites.

Identification of components of an aphid wing base was based on Snodgrass [20] (Figure 1) and Matsuda [17]. The studied sclerite is in the prealar position but is located closer to the prescutum, in an antero–lateral position, homologous with the tegula in *Psylla* and *Aphis* [17,33,34].

## 3. Results

**Figure 2:**
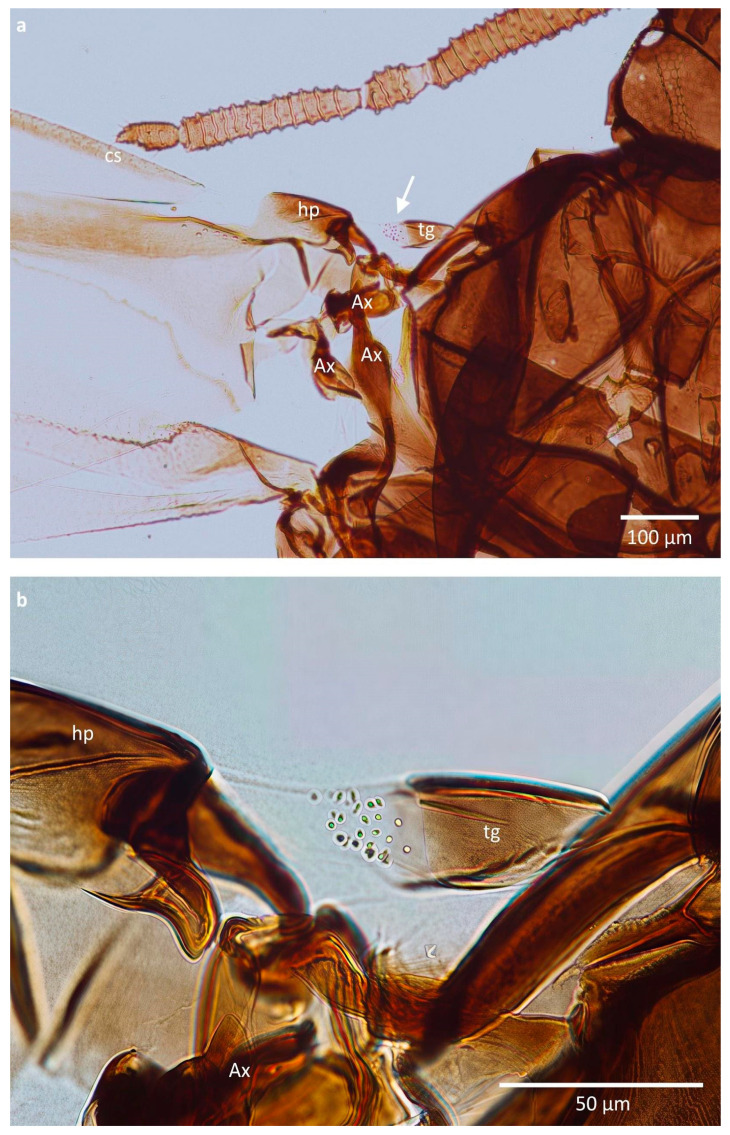
Wing base of *Tetraneura nigriabdominalis*: (**a**) placement of studied region, (**b**) sense pegs as visible in light microscopy. Abbreviations: cs—costal vein, hp—humeral plate (basicosta), tg—tegula, Ax—axillary sclerite.

The tegulae in the studied specimens consisted of a sclerotised and hairless proximal part and a membranous distal part, covered with very small, barely visible in light microscopy, cuticular pegs (Figure 2a). It was difficult to perform an exact counting of these pegs due to their variable size; however, their number seemed to oscillate between 10 and 32 per tegula, depending on the species and morph (Figure 2b, Table 1). The greatest difference in the number of pegs was observed in *E. ulmi*, where sexuparous females had only 14.11 of them on average (10–19), while their alate fundatrigeniae had as many as 27.40 on average (22–32). The difference between fundatrigenia and sexupara of *T. ulmi* was very small.

**Table 1:** Number of cuticular pegs in studied specimens visible in light microscopy.

Species Name	Number of Studied Specimens	Number of Tegulas Available for Study	Number of Pegs on Tegula (±SD)
*Tetraneura ulmi*, fundatrigenia	2	3	17.33 ± 2.62
*Tetraneura ulmi*, sexupara	5	8	16.75 ± 4.18
*Tetraneura nigriabdominalis*, sexupara	3	5	22.60 ± 2.87
*Eriosoma ulmi*, fundatrigenia	3	5	27.40 ± 3.32
*Eriosoma ulmi*, sexupara	9	9	14.11 ± 2.60

**Figure 3:**
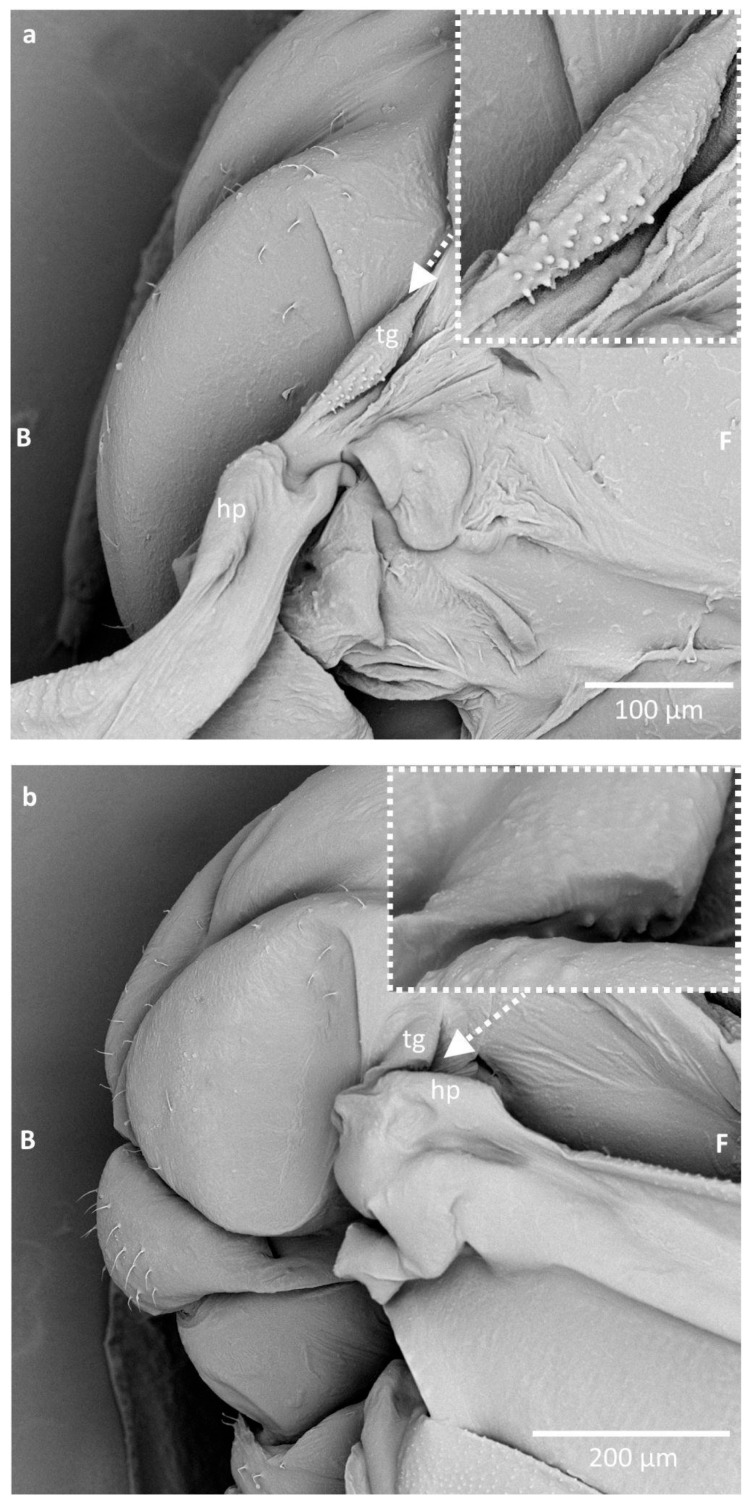
The position of the studied structures during the wing movement in *Eriosoma ulmi*: (**a**) the position of the wing during the upstroke; (**b**) the position of the wing during the downstroke. Abbreviations: B—back of the body, F—front of the body, tg—tegula, hp—humeral plate.

In SEM, these pegs were more conspicuous; however, mounting the specimen was causing a bending of the tegula, so it was difficult to obtain a full view of the whole field covered with these structures. It seems that during bending of the wing, the part of the tegula with pegs is matching the cuticular arch beneath the humeral plate, at its proximal edge (Figure 3).

**Figure 4:**
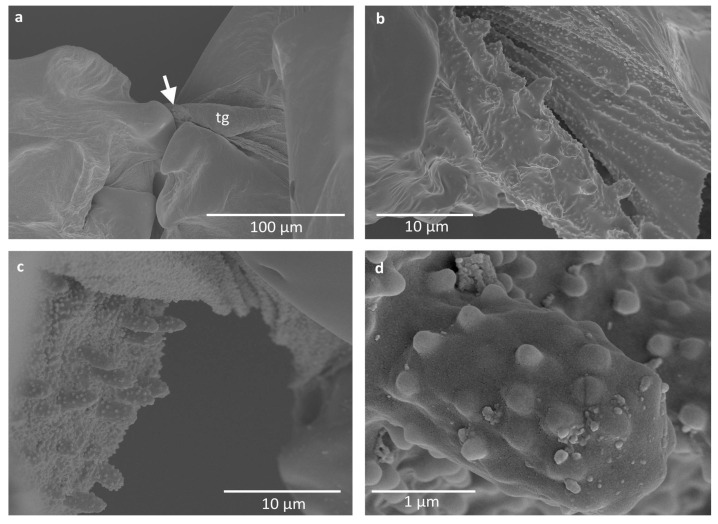
Scanning electron microscopy imaging of studied structures in *Eriosoma ulmi*: (**a**) general view; (**b**,**c**) close-up on the field with sensory pegs in different specimens; (**d**) close-up on a single peg. Abbreviation: tg—tegula.

**Figure 5:**
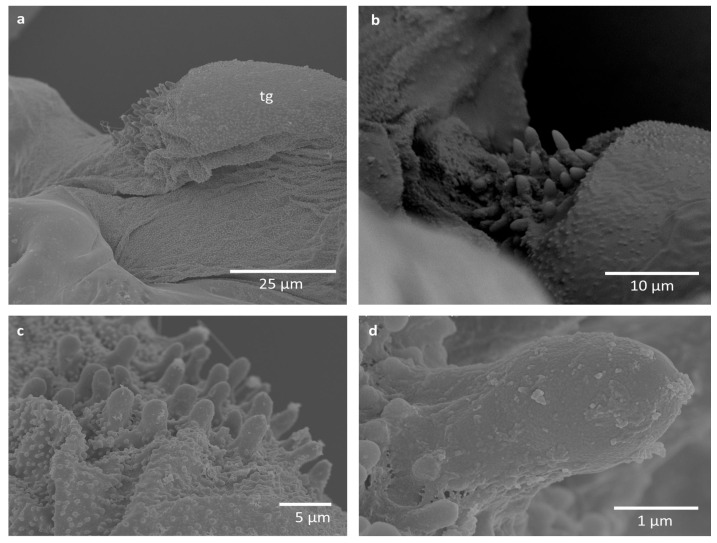
Scanning electron microscopy imaging of studied structures in *Tetraneura nigriabdominalis*: (**a**) general view; (**b**,**c**) close-up on the field with sensory pegs in different specimens; (**d**) close-up on a single peg. Abbreviation: tg—tegula.

**Figure 6:**
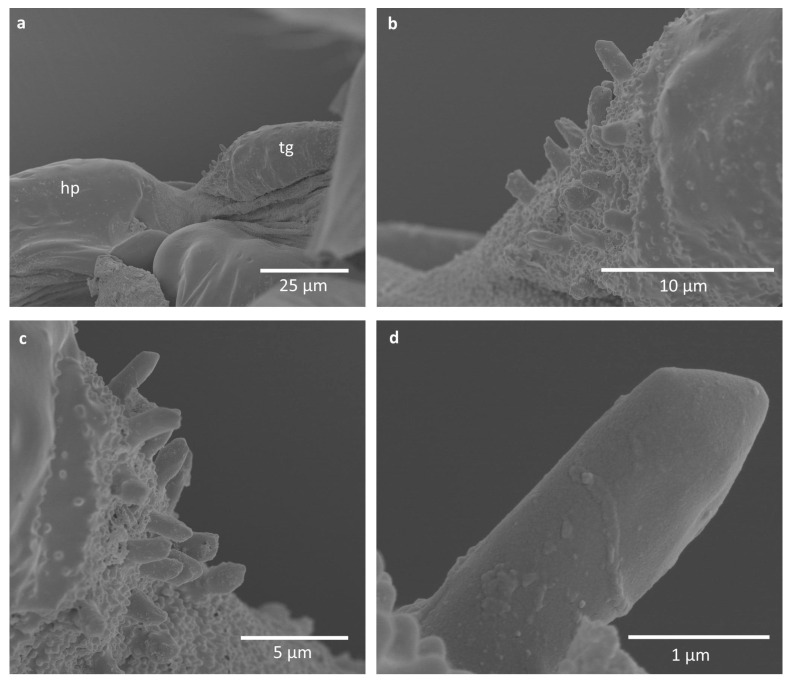
Scanning electron microscopy imaging of studied structures in *Tetraneura ulmi*: (**a**) general view; (**b**,**c**) close-up on the field with sensory pegs in different specimens; (**d**) close-up on a single peg. Abbreviations: hp—humeral plate, tg—tegula.

Some difficulties in proper imaging were also caused by the wax covering of the studied specimens, although minute nodes on the pegs and around them seem not to be wax coverings, as they are merged with the surface (Figure 4, Figure 5 and Figure 6). They occur on pegs in *E. ulmi* (Figure 4d), while the pegs of *Tetraneura* spp. were smooth and free of any protuberances (Figure 5d and Figure 6d), although the area around them was also covered by such nodes (Figure 5a–c and Figure 6a–c).

The studied pegs were very small, being ca. 3–4 μm high and 1–2 μm in diameter, although their size was not uniform (Table 2), with pegs in *T. ulmi* slightly shorter than in *T. nigriabdominalis* and *E. ulmi*. No trace of any cuticular socket around the peg bases was visible; they seem to be protruding directly from the cuticle.

## 4. Discussion

In insects, the tegula is known to possess sensory setae, which may serve various functions. such as controlling flight muscles in locusts, stridulation in Lepidoptera, and location of different types of sensilla and chordotonal in Acrididae and Calliphoridae [18,21,22,23,24].

The observed cuticular pegs are positioned on the distal part of the tegula, less sclerotised and very short. They do not seem to be homologous with sensory setae, as there were no observable sockets, typical of setae, especially those serving as sensillary organs. They also do not resemble any of the chemoreceptors, as they do not possess pores on the surface. Previously studied tegular sensilla of other insect taxa showed the presence of mostly campaniform sensilla, hair-like sensilla, and chordotonal organs [18,23,24]. The observed pegs in the presently studied insects are rather cuticular knobs, very short and scattered over a relatively small area. They do not meet the criteria of either campaniform or hair-like sensilla—they are neither flat oval discs, nor tapered hairs. It may only be hypothesized that they are endings of subcuticular scolopidia, but proving that would require cross sections. Since the first phase of this study included morphological examination of the structures, light and scanning microscopes were used for this purpose. Research is ongoing and the next phase will be the collection of the specimens and glutaraldehyde fixation, which will allow for cross sections to be studied. It is known that a single chordotonal organ in an insect’s tegula may contain up to 30 scolopidia, each of them connected to a single cuticular knob [24,25]. So far, such structures have not been described at the wing bases of aphids. It must also be noted that their positioning, as seen in the microscopic slide, is unrealistic because the whole thorax is strongly pressed under the glass cover, and thus, the tegula extrudes significantly from the thorax. In the case of the specimens of scanning electron microscopy, the tegula is slightly closer to the pleural part of the thorax, yet the part with pegs is extruding from the thorax.

These structures might be interpreted as a form of stridulatory apparatus, although their arrangement is irregular and their height is uneven. It seems that, during bending of the wing, the pegs of the tegula (functioning as a sort of a plectrum) match the concave sclerotised arch within the proximal part of the humeral plate (functioning as stridulitrum) (Figure 3). The presence of stridulatory apparatus is well documented in Hemiptera [35,36], including in aphids (e.g., *Toxoptera aurantii* which stridulates by rubbing with cuticular pegs of the hind tibiae on the postero-marginal part of abdomen, where rows of cuticular denticles are located [37]) but not around the wings of aphids. Additionally, no hearing organ (tympanal organ) is known in aphids, but mechanoreceptors in the form of setae could detect sound as an air movement (sound waves). Nevertheless, it does not seem that the described structures may cause stridulation, as these pegs are not rubbing the cuticular ridge, and the tegula in this part rather fills in the concave space under the humeral plate. The pegs are also rather on the soft, membranous part of the tegula, whereas a stridulatory apparatus typically requires rigid and solid structuring. Additionally, the stridulatory apparatus in other hemipterans, either the plectrum or the stridulitrum, is rather linear, while in the present case such an arrangement is not observed.

The alternative hypothesis is that these cuticular pegs have some mechanical function either during flight or when the wings are reposed, rubbing the humeral plate or axillary sclerites. However, they are extremely small, and it is difficult to determine their function. The area covered with pegs is very rough, due to the presence of larger pegs and a lot of very small cuticular projections (Figure 4c,d, Figure 5c,d and Figure 6c). It seems that this may have some mechanical properties, perhaps proprioceptive, as a part of the chordotonal organ, regulating the upstroke of the forewing [21] or its bending, as a connective chordotonal organ [11]. Additional research must concern the tegula in other aphid subfamilies, comprising observations in vivo, as well as detailed transmission electron microscopy observations.

## 5. Conclusions

The tegula of the forewings in alate morphs of aphids in the subfamily Eriosomatinae is in its membranous part, covered with cuticular pegs of unknown function. It seems most probable that they serve a proprioceptive function, as a part of the chordotonal organ, to regulate the muscles bending the wings. Further studies including other aphid subfamilies are needed to clarify the role of this structure.

## Figures and Tables

**Table 2:** Characteristics of cuticular pegs of studied specimens as visible in scanning electron microscopy.

Species Name	Average Height of Cuticular Pegs (µm) (±SD)	Average Width of Cuticular Pegs (µm) (±SD)	External Appearance
*Eriosoma ulmi*	3.83 ± 0.64	1.74 ± 0.23	Covered with cuticular grains
*Tetraneura ulmi*	2.92 ± 0.36	1.26 ± 0.20	Smooth
*Tetraneura nigriabdominalis*	3.69 ± 0.38	1.39 ± 0.21	Smooth

## Data Availability

The original contributions presented in this study are included in the article. Further inquiries can be directed to the corresponding author.
